# The non-classical immune checkpoint HLA-G: a regulatory master switch governing tolerance, evasion, and translational frontiers

**DOI:** 10.3389/fonc.2026.1761266

**Published:** 2026-02-19

**Authors:** Huan Liu, Qiong Li, Junli Li, Ying Xue, Xiaofei Xue, Pingping Xu

**Affiliations:** School of Smart Health Science & Technology, Zhengzhou Health College, Zhengzhou, Henan, China

**Keywords:** cancer immunotherapy, HLA-G, immune checkpoint, immune tolerance, immune evasion

## Abstract

Human Leukocyte Antigen G (HLA-G), a non-classical MHC Class I molecule, plays a pivotal role in immune regulation, particularly in reproductive immunology. It functions as an immune checkpoint by interacting with inhibitory receptors such as LILRB1 (ILT2/CD85j) and LILRB2 (ILT4/CD85d) on both innate and adaptive immune cells. HLA-G is crucial for maintaining immune tolerance, with its expression by extravillous trophoblasts being essential for fetal survival and establishing materno-fetal immune privilege. In transplantation, HLA-G promotes graft acceptance and serves as a positive prognostic marker. However, its tolerogenic function is exploited by malignant cells to evade immune detection, inhibiting cytotoxic T-lymphocyte (CTL) and NK cell functions, inducing regulatory Treg cells, and remodeling the tumor microenvironment (TME). Elevated HLA-G expression correlates with poor prognosis in various cancers, with a meta-analysis showing a Hazard Ratio for mortality of 2.09. HLA-G’s soluble isoforms (sHLA-G) and exosome-mediated HLA-Gev (HLA-G-bearing extracellular vesicles) transfer are emerging as potential liquid biopsy markers. Targeting the HLA-G/ILT axis is a promising therapeutic strategy, with clinical trials underway using anti-HLA-G antibodies (e.g., TTX-080) and anti-LILRB1 antibodies (e.g., BND-22), often combined with PD-1/PD-L1 inhibitors. Additionally, HLA-G agonists or engineered cells are being explored for inducing tolerance in autoimmune diseases and transplantation.

## 1 Introduction

### 1.1 The MHC-I family: functional divergence of classical and non-classical molecules

The Major Histocompatibility Complex (MHC) gene region encodes proteins that are foundational to immune surveillance and self/non-self discrimination. These proteins are broadly categorized into classical (MHC-Ia) and non-classical (MHC-Ib) molecules, which possess fundamentally divergent functions (1).

Classical MHC-I molecules, namely HLA-A, HLA-B, and HLA-C in humans, are defined by their high degree of polymorphism and their ubiquitous expression across almost all nucleated cells. Their primary and canonical function is to present a vast repertoire of intracellularly derived antigenic peptides to the T-cell receptors (TCRs) of CD8+ cytotoxic T-lymphocytes (CTLs) (2). This interaction is the principal mechanism by which the adaptive immune system recognizes and eliminates virus-infected or malignantly transformed cells.

In contrast, the non-classical MHC-Ib molecules, which include HLA-G, HLA-E, and HLA-F, represent a more functionally diverse and specialized group (3). They are characterized by limited polymorphism, restricted and often inducible tissue expression, and a broader range of immunoregulatory roles that extend beyond conventional antigen presentation (4). A key functional divergence is that while MHC-Ia molecules are the primary ligands for T-cell activation, MHC-Ib molecules like HLA-G serve to mediate inhibitory or, in some cases, activating stimuli, particularly for Natural Killer (NK) cells of the innate immune system (5). HLA-G, the focus of this review, epitomizes this immunoregulatory role, acting as a potent ligand for inhibitory receptors that bridge both innate and adaptive immunity (6).

### 1.2 The molecular uniqueness of HLA-G: limited polymorphism, multiple isoforms, and key receptors (LILRB1/LILRB2)

HLA-G possesses several unique molecular features that distinguish it from its classical counterparts and define its specialized function. Firstly, the HLA-G gene exhibits remarkably low polymorphism in its coding region (7). This evolutionary conservation is in stark contrast to the hypervariable nature of classical HLA genes and is critical for HLA-G to function as a “universal” inhibitory ligand, capable of engaging its receptors on the immune cells of genetically diverse individuals.

Secondly, the HLA-G primary transcript undergoes complex alternative splicing to generate at least seven distinct protein isoforms, a level of diversity not seen in classical HLA molecules (8). These isoforms are structurally and functionally heterogeneous, broadly classified into four membrane-bound (mHLA-G) isoforms (HLA-G1, -G2, -G3, and -G4) and three soluble (sHLA-G) isoforms (HLA-G5, -G6, and -G7) (9). The full-length HLA-G1 protein and its soluble counterpart, HLA-G5, are the most extensively studied (10).

Thirdly, and most central to its function, HLA-G exerts its profound immunomodulatory effects by serving as a ligand for a specific set of inhibitory receptors expressed on immune effector cells (11). The most critical of these are the Immunoglobulin-Like Transcript (ILT) receptors, now standardized as Leukocyte Immunoglobulin-Like Receptors (LILRs) (11). The most critical of these are: LILRB1 (ILT2/CD85j): This receptor is broadly expressed on B cells, monocytes, dendritic cells (DCs), and distinct subsets of T cells and NK cells (8). It recognizes both classical MHC-I molecules and HLA-G, though it binds HLA-G with significantly higher affinity (12, 13). LILRB2 (ILT4/CD85d): The expression of this receptor is more restricted to the myeloid lineage, including monocytes, macrophages, and dendritic cells (8). Unlike LILRB1, LILRB2 demonstrates a preferential binding to HLA-G over classical MHC-I molecules (12).

The signaling axis formed by the interaction of HLA-G with LILRB1 and LILRB2 is the central molecular pathway for HLA-G-mediated immune suppression and its function as a non-classical immune checkpoint (14).

### 1.3 The central thesis: HLA-G as a “dual-role” master switch in immune regulation

The highly restricted expression pattern of HLA-G under normal physiological conditions—confined to sites like the placenta, thymus, and cornea—points to its dedicated role as a guardian of immune privilege (15). In these “sanctuary” tissues, its function is not only beneficial but essential for maintaining immune tolerance and preventing autoimmune or allo-immune-mediated pathology (16).

However, this potent tolerogenic function possesses a pathological “dark side.” HLA-G is frequently neo-expressed or aberrantly upregulated in a wide array of pathological conditions, most notably in solid and hematological malignancies and during chronic viral infections (17). In these disease states, HLA-G expression is no longer a protective physiological mechanism but is instead hijacked as a sophisticated tool for immune evasion (18). This aberrant expression allows tumor cells and virus-infected cells to “disarm” the host immune response by engaging inhibitory receptors, leading directly to disease progression, metastasis, and poor clinical outcomes (19).

**Figure 1:**
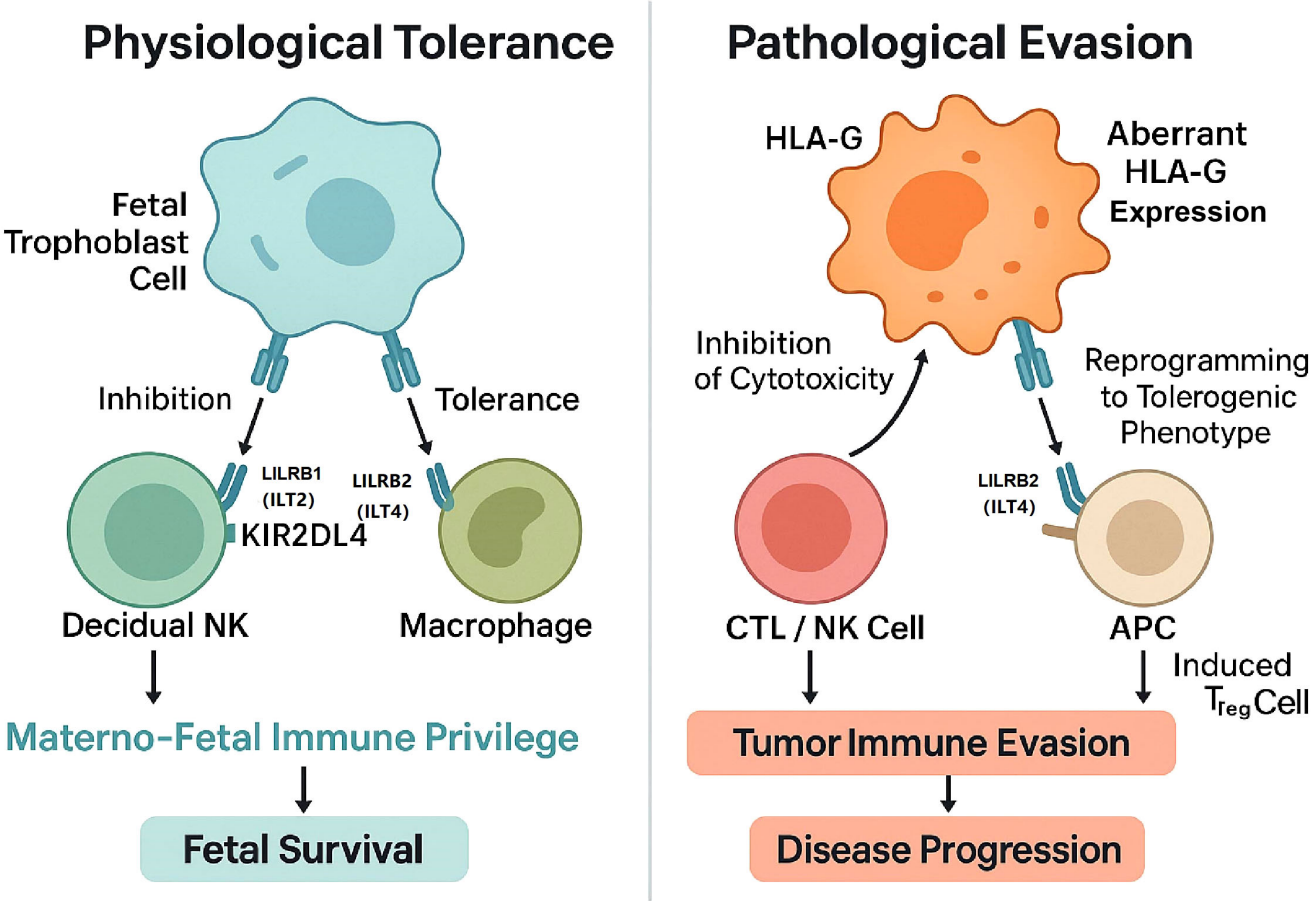
The dual-role master switch of HLA-G in immune regulation. This schematic illustrates the divergent outcomes of HLA-G signaling depending on the biological context. (Left) Physiological Tolerance: Under normal conditions, such as the materno-fetal interface, HLA-G expressed by fetal trophoblast cells interacts with inhibitory receptors LILRB1 and KIR2DL4 on dNK cells, and LILRB2 on macrophages. This interaction actively establishes materno-fetal immune privilege, neutralizing potential maternal immune attacks and ensuring fetal survival. (Right) Pathological Evasion: In the context of malignancy, tumor cells exploit this mechanism through aberrant or *de novo* HLA-G expression. By engaging LILRB1 on CTLs and NK cells, HLA-G inhibits direct cytotoxicity, while engagement of LILRB2 on Antigen-Presenting Cells (APCs) induces a tolerogenic phenotype and promotes the expansion of induced Regulatory T cells (Tregs). This "onco-fetal" mimicry shields the tumor from immune surveillance, ultimately driving disease progression.

This review is structured around this central thesis: HLA-G is a “dual-role” molecule, acting as a molecular master switch that can be physiologically engaged to promote tolerance or pathologically exploited by disease to drive evasion (**Figure 1**).

### 1.4 Structure and objectives of this review

In accordance with the central thesis, this review will first dissect the fundamental molecular biology of HLA-G, including its complex isoform structure and the regulatory networks that govern its expression (11). It will then explore the two faces of its dual function: its essential role in establishing and maintaining physiological tolerance in pregnancy, transplantation, and immune-privileged sites, followed by its detrimental role in pathological immune evasion in cancer and chronic infections. Finally, we will survey the rapidly advancing translational frontiers, critically evaluating HLA-G as a diagnostic biomarker and as a next-generation therapeutic target, before concluding with a discussion of the major challenges and future perspectives for the field.

## 2 Biological foundations and regulatory network of HLA-G

### 2.1 Molecular architecture: generation and functional differences of membrane-bound (G1-G4) and soluble (G5-G7) isoforms

**Figure 2:**
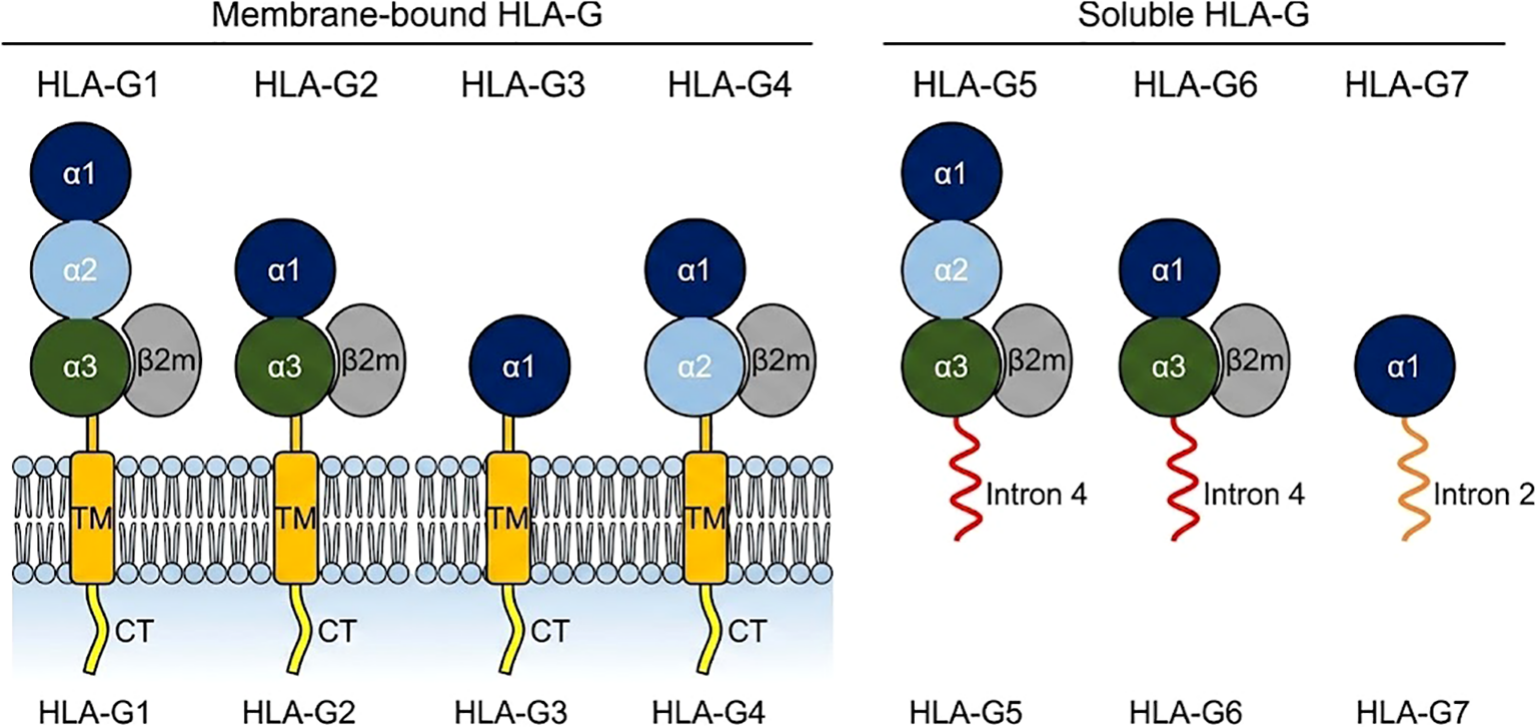
Schematic representation of the seven HLA-G protein isoforms. This figure illustrates the structural diversity generated by alternative splicing of the HLA-G primary transcript. **(A)** Membrane-bound isoforms (HLA-G1 to HLA-G4): These isoforms are anchored to the cell membrane via a transmembrane domain and a cytoplasmic tail. HLA-G1 is the full-length molecule comprising α-1, α-2, and α-3 domains associated with β-2-microglobulin (β-2m). HLA-G2, HLA-G3, and HLA-G4 are truncated isoforms lacking specific extracellular domains (α-2, α-2/α-3, and α-3, respectively). **(B)** Soluble isoforms (HLA-G5 to HLA-G7): These isoforms lack transmembrane and cytoplasmic domains and are secreted into the extracellular space. HLA-G5 and HLA-G6 retain intron 4-encoded amino acids at the C-terminus (shown in red), which confer solubility. HLA-G7 retains intron 2 (shown in orange) and is the only isoform that does not bind β-2m. (Note: Color coding distinguishes specific domains and tails).

The functional versatility of HLA-G is encoded in its molecular architecture, which is defined by the seven distinct protein isoforms generated via alternative splicing of the HLA-G primary transcript (20) (**Figure 2**).

Membrane-bound (mHLA-G) Isoforms (**Table 1**):

**Table 1:** The seven isoforms of HLA-G.

Isoform	Form (membrane/soluble)	Molecular structure (exons/introns)	Association with β2m	Key structural feature	Physiological/pathological role	References
HLA-G1	Membrane-bound	Exons 1-8	Yes	Full-length protein (α1, α2, α3, TM)	Canonical membrane-bound isoform mediating materno-fetal tolerance via LILRB1/2; highly expressed in tumors for immune evasion.	(9, 11, 21)
HLA-G2	Membrane-bound	Exons 1, 2, 4, 5-8	Yes	Lacks α2 domain (Exon 3 skipped)	Homodimer-forming isoform found in placenta and tumors; may exhibit distinct receptor affinity due to α2 deletion.	(20, 22)
HLA-G3	Membrane-bound	Exons 1, 2, 5-8	Yes	Lacks α2, α3 domains (Exons 3, 4 skipped)	Highly truncated; detected in colorectal cancer and other malignancies; signaling capacity remains less defined.	(20, 22)
HLA-G4	Membrane-bound	Exons 1, 2, 3, 5-8	Yes	Lacks α3 domain (Exon 4 skipped)	Lacks α3 domain (LILRB2 binding site), suggesting a unique regulatory role in immune signaling.	(20)
HLA-G5	Soluble	Exons 1-4, (Intron 4)	Yes	Full-length extracellular + 21aa soluble tail	Primary soluble isoform mediating systemic suppression; associated with graft acceptance but poor cancer prognosis.	(23–25)
HLA-G6	Soluble	Exons 1, 2, 4, (Intron 4)	Yes	α2-lacking extracellular + 21aa soluble tail	Soluble counterpart to G2; detected in stem cells and cancer; functional role under investigation.	(22, 26)
HLA-G7	Soluble	Exon 1, 2, (Intron 2)	No	α1 domain only + soluble tail	Soluble isoform lacking β2m binding; biological function remains largely uncharacterized.	(26, 27)

HLA-G1: This is the canonical, full-length MHC-I protein, structurally homologous to classical HLA molecules. It consists of the α1, α2, and α3 extracellular domains, a transmembrane domain (encoded by exon 5), and a cytoplasmic tail (encoded by exons 6-8). It non-covalently associates with β2-microglobulin β2m (9). HLA-G2: A truncated isoform generated by the splicing (skipping) of exon 3, which encodes the α2 domain (20). HLA-G3: A further truncated isoform lacking both the α2 and α3 domains, resulting from the splicing of both exon 3 and exon 4 (20). HLA-G4: An isoform that retains the α1 and α2 domains but lacks the α3 domain due to the skipping of exon 4 (20).

Soluble (sHLA-G) Isoforms:

HLA-G5: This is the primary soluble isoform and is structurally analogous to HLA-G1. It is generated when intron 4 is retained in the mature mRNA. This intron contains a premature stop codon, which prevents the translation of the downstream transmembrane domain (23). This process results in a protein containing the full α1-α3 extracellular domain associated with β2m, but with a unique 21-amino-acid C-terminal tail that confers solubility (26).

HLA-G6: The soluble counterpart to HLA-G2, this isoform lacks the α2 domain and is also rendered soluble by the retention of intron 4 (22). HLA-G7: A highly truncated soluble isoform consisting only of the α1 domain, resulting from the retention of intron 2 (27).

These structural variations dictate receptor engagement. Not all isoforms engage receptors equally. For instance, dimeric forms of HLA-G (linked via Cys42-Cys42 disulfide bonds) exhibit a significantly higher affinity for LILRB1 and LILRB2 compared to their monomeric counterparts (28, 29). Specifically, the dimerized HLA-G molecule presents an accessible binding site that favors LILRB1 interaction, effectively increasing the avidity of the immune checkpoint signal (28). Furthermore, truncated isoforms lacking the α3 domain (such as HLA-G4) may fail to bind LILRB2 efficiently, as the α3 domain is a critical contact site for this receptor (30). Thus, the specific isoform profile of a tissue directly shapes its immunomodulatory potential.

### 2.2 A tightly regulated gene: transcriptional, epigenetic (methylation), and post-transcriptional (miRNA) control

The restricted, “on-demand” expression of HLA-G is maintained by a multi-layered and highly sophisticated regulatory network. While its coding region is conserved, the HLA-G gene’s regulatory regions—specifically the 5’ upstream regulatory region (URR) and the 3’ untranslated region (UTR)—are, in contrast, highly polymorphic. This suggests that evolutionary selective pressure has favored the ability to modulate expression levels of HLA-G rather than alter its core inhibitory function (7, 31, 32).

Transcriptional and Epigenetic Control: In most normal, healthy tissues, the HLA-G gene is transcriptionally silent. This silencing is often enforced by epigenetic mechanisms, primarily DNA hypermethylation at CpG islands within the promoter region (33). In pathological contexts, such as cancer, the *de novo* expression of HLA-G is frequently linked to the loss of this silencing, often through promoter demethylation. Furthermore, polymorphisms within the 5’ URR can alter the binding affinity of key transcription factors, thereby dictating the basal and inducible levels of HLA-G transcription (20, 34).

Post-Transcriptional (miRNA) Control: The 3’ UTR of the HLA-G mRNA is a critical hub for post-transcriptional regulation, acting as a target for non-coding microRNAs (miRNAs) (35).

miR-148a and miR-152: A significant body of evidence has identified miR-148a and miR-152 as key negative regulators of HLA-G. These miRNAs bind to specific sites in the HLA-G 3’ UTR, triggering mRNA degradation and/or translational repression, thereby silencing HLA-G protein expression (36).

Tissue-Specific Regulation: This mechanism is fundamental to HLA-G’s tissue-specific expression. The physiologically high expression of HLA-G in the placenta, for instance, is not only due to transcriptional activation but also to the fact that the placenta lacks high-level expression of miR-148a and miR-152. This low-miRNA environment permits the HLA-G mRNA to remain stable and be efficiently translated, which is essential for materno-fetal tolerance (32).

**Figure 3:**
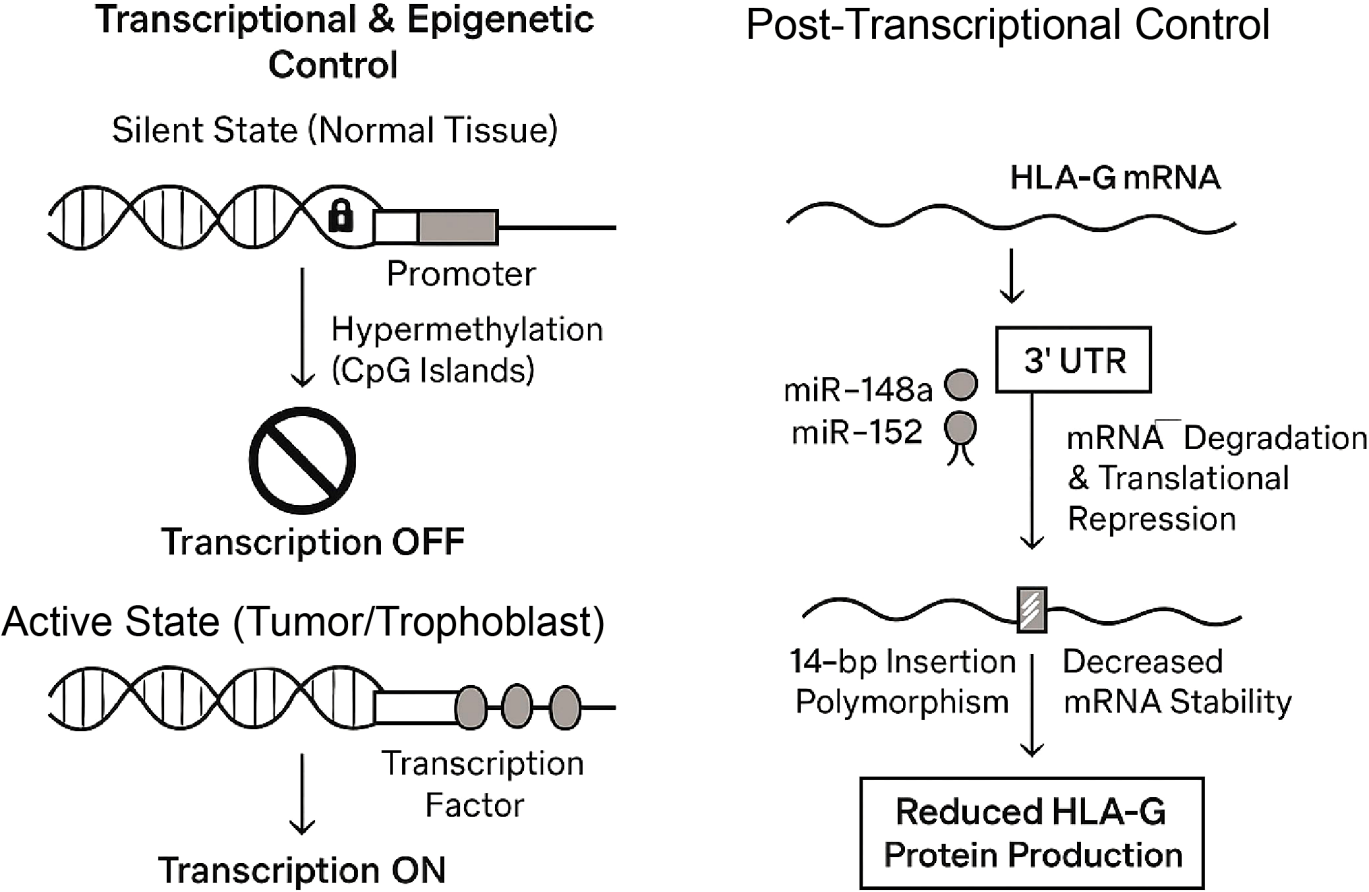
Multi-layered regulatory network of HLA-G expression.The expression of HLA-G is tightly controlled at multiple levels.Left panel (Transcriptional & Epigenetic Control): In normal tissues, the HLA-G gene is kept in a "Silent State" via DNA hyper-methylation of CpG islands in its promoter region, which prevents transcription. In tumor cells and trophoblasts, this promoter is demethylated, allowing transcription factors to bind and switch transcription "ON" (Active State). Right panel (Post-Transcriptional Control): The stability and translation of HLA-G mRNA are regulated by its 3' Untranslated Region (UTR). MicroRNAs, specifically miR-148a and miR-152, bind to the 3' UTR to trigger mRNA degradation and translational repression. Furthermore, the 14-bp insertion polymorphism (rs371194629) within the 3' UTR is linked to decreased mRNA stability. Both mechanisms result in reduced HLA-G protein production.

Impact of Regulatory Polymorphisms: Genetic variations within these regulatory regions directly impact the level of HLA-G protein expression, linking an individual’s genotype to their immune-regulatory potential. The most extensively studied example is the 14-base pair (bp) insertion/deletion polymorphism (rs371194629) located in the 3’ UTR (31, 37). The presence of the 14-bp insertion allele has been functionally linked to lower mRNA stability and, consequently, reduced HLA-G protein production. This is thought to occur because the insertion sequence alters the secondary structure of the mRNA, potentially affecting its stability or its processing via alternative splicing of the 3’ UTR itself (**Figure 3**) (38).

### 2.3 The HLA-G/LILRB signaling axis: molecular mechanisms of immune inhibition

The interaction between HLA-G and its receptors triggers distinct intracellular signaling cascades depending on the cell type involved, effectively reprogramming the immune microenvironment (11).

Inhibition of Cytotoxicity (via LILRB1): In effector cells such as NK cells and CD8+ T cells, the engagement of LILRB1 by HLA-G initiates a direct inhibitory program. Upon ligand binding, the Immunoreceptor Tyrosine-based Inhibitory Motifs (ITIMs) located within the cytoplasmic tail of LILRB1 undergo phosphorylation (12). This modification creates a docking site for the protein tyrosine phosphatases SHP-1 and SHP-2. Once recruited, these phosphatases dephosphorylate key signaling molecules involved in the activation pathways (such as ZAP70 or Syk), effectively terminating the “activation” signal (12, 39). This process directly suppresses granule exocytosis, cytokine production, and cell proliferation, thereby neutralizing the cytotoxic function of the immune effector cell (40).

**Figure 4:**
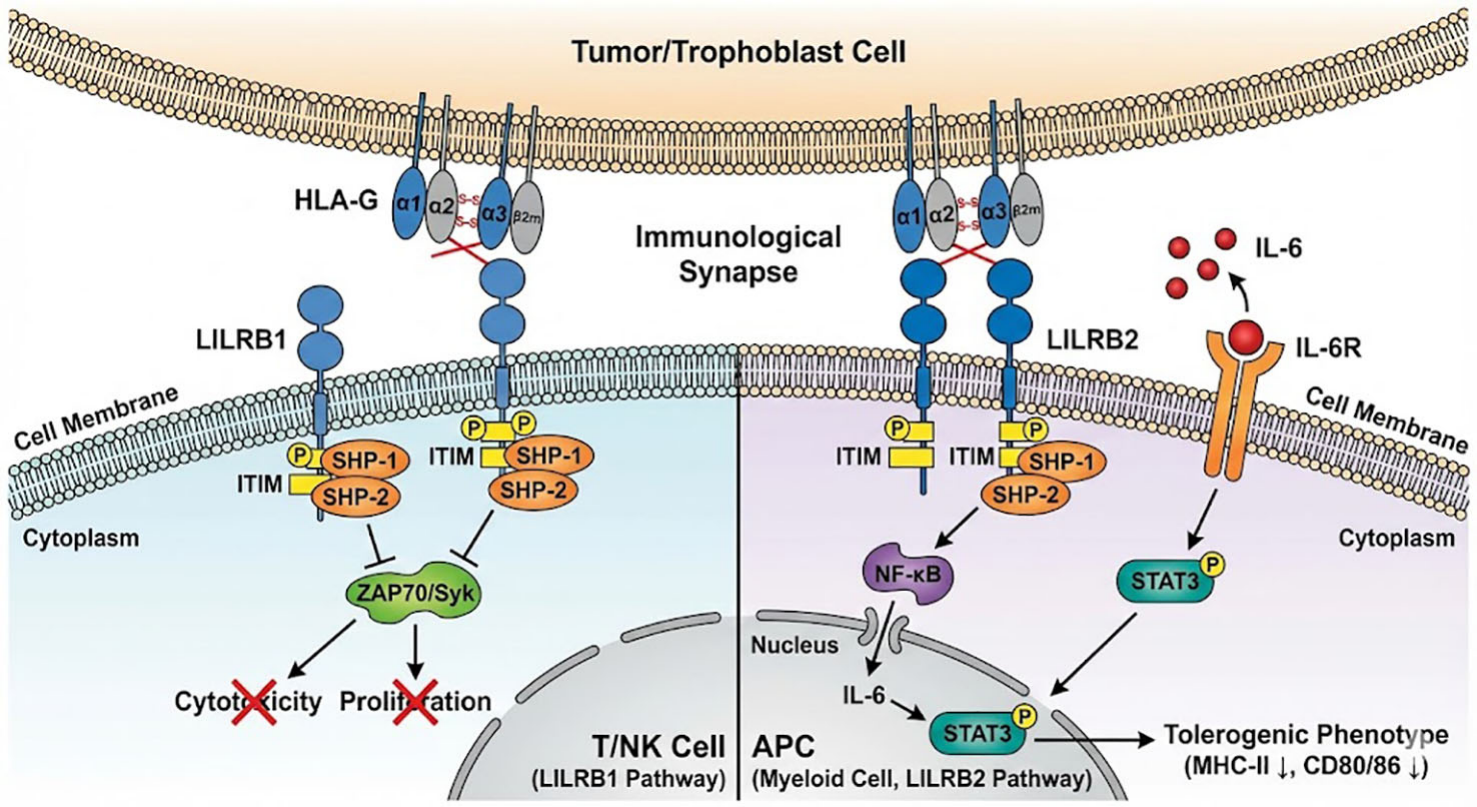
The molecular mechanisms of HLA-G-mediated immune suppression. The diagram illustrates the distinct signaling pathways triggered by HLA-G interaction with its primary receptors on different immune cells. **(A)** Inhibition of Cytotoxicity: On the left, HLA-G expressed on tumor or trophoblast cells binds to the LILRB1 (ILT2) receptor on the membrane of T cells or NK cells. This interaction triggers the phosphorylation of intracellular Immunoreceptor Tyrosine-based Inhibitory Motifs (ITIMs), leading to the recruitment of SHP-1 and SHP-2 phosphatases. These phosphatases dephosphorylate key signaling molecules, thereby blocking activating pathways and inhibiting cell cytotoxicity and proliferation. **(B)** APC Reprogramming: On the right, HLA-G binds to the LILRB2 (ILT4) receptor on the membrane of myeloid Antigen-Presenting Cells (APCs). This engagement initiates a signaling cascade involving SHP-1/2 recruitment and non-canonical NF-κB activation, which promotes IL-6 secretion. Secreted IL-6 acts in an autocrine/paracrine loop to activate STAT3, driving the APC toward a tolerogenic phenotype characterized by arrested maturation (downregulation of MHC-II and CD80/86) and the induction of regulatory T cells (Tregs).

Reprogramming of Antigen-Presenting Cells (via LILRB2): In myeloid APCs, the engagement of LILRB2 by HLA-G induces a more complex functional shift known as “tolerogenic reprogramming” (41). Unlike the canonical inhibitory pathway, the recruitment of SHP-2 by LILRB2 in dendritic cells and macrophages has been linked to the paradoxical activation of the NF-κB signaling pathway (42).This activation drives the transcription and secretion of Interleukin-6 (IL-6) (43).The secreted IL-6 then acts in an autocrine or paracrine manner to bind IL-6 receptors on the APC surface, triggering the phosphorylation of STAT3 (44).Constitutive STAT3 activation locks the APC into a tolerogenic state, characterized by the downregulation of MHC Class II and costimulatory molecules (CD80/86) and resistance to maturation signals (45). Consequently, these “reprogrammed” APCs not only fail to prime effector T cells but actively promote the differentiation of inducible regulatory T cells (iTregs) (46). Thus, the HLA-G/LILRB2 axis acts as a sophisticated switch to convert antigen-presenting cells from immunogenic to tolerogenic, actively shaping a suppressive microenvironment (**Figure 4**, **Table 2**).

**Table 2:** Key receptors of the HLA-G axis.

Receptor	Alternative name(s)	Primary cellular expression	Signaling motif	Key downstream effect of HLA-G binding	References
LILRB1	LILRB1, CD85j	B cells, Monocytes, DCs, T cells, NK cells	ITIM	Broad immune inhibition; inhibits cytolysis of T/NK cells.	(8, 12, 39, 47)
LILRB2	LILRB2, CD85d	Monocytes, Macrophages, Dendritic Cells	ITIM	Induces tolerogenic APC phenotype via IL-6/STAT3 pathway.	(8, 12, 42, 45)
KIR2DL4	CD158d	NK cells (esp. decidual)	Complex (Inhibitory/Activating)	Modulates NK cell function; central to materno-fetal tolerance.	(41, 48)

## 3 Physiological functions of HLA-G: the cornerstone of immune tolerance

### 3.1 Materno-fetal tolerance: trophoblast expression and the establishment of immune privilege

The canonical and most well-understood physiological role of HLA-G is in human pregnancy, where it is the central mediator of materno-fetal tolerance (21). The fetus is a semi-allograft, expressing paternal antigens that should, by all immunological rules, be recognized as “non-self” and rejected by the maternal immune system. This rejection is actively prevented.

The mechanism for this tolerance is localized at the materno-fetal interface. Fetal-derived extravillous trophoblast (EVT) cells, which are invasive and migrate into the uterine wall (decidua), come into direct contact with maternal immune cells. These EVT cells have a unique and specialized MHC profile: they do not express the classical, highly polymorphic HLA-A and HLA-B molecules, which would present paternal peptides and trigger a CTL response. Instead, they uniquely and abundantly express HLA-G (21, 49).

This trophoblast-expressed HLA-G (both membrane-bound and soluble) interacts with the array of inhibitory receptors (LILRB1, LILRB2, and KIR2DL4) on the large population of maternal immune cells in the decidua, particularly decidual NK (dNK) cells and macrophages (48). This engagement neutralizes the cytotoxic potential of dNK cells, modulates macrophages towards a supportive (M2) phenotype, and protects the semi-allogeneic fetus from immune attack (15). This process actively establishes the placenta as a site of profound, HLA-G-mediated immune privilege.

The clinical significance of this tolerance mechanism is distinctively underscored by its disruption in pathological pregnancies. Emerging evidence suggests that the dysregulation of the HLA-G inhibitory axis is a critical driver of recurrent pregnancy loss (RPL) and infertility. Specifically, recent clinical reviews have highlighted that ‘immunological mismatch’ and defects in maternal-fetal compatibility are central to the pathogenesis of RPL (50). This pathology is often mediated by functional imbalances in dNK cells and T cell subsets (51), as well as specific alloimmune mechanisms that disrupt the delicate pro- and anti-inflammatory balance required for implantation (52). At the molecular level, the regulatory function of HLA-G in early pregnancy is exerted through complex signaling interactions with receptors on immune cells, particularly NK cells (53). Crucially, mechanistic studies—including those investigating tumor resistance models—have further elucidated how HLA-G engagement with receptors like KIR2DL4 can orchestrate immune escape and cytokine signaling (e.g., via JAK2/STAT1 pathways), offering translational insights that are highly relevant to understanding immune privilege at the fetal interface (54).

### 3.2 Transplantation immunology: HLA-G as a predictive biomarker and therapeutic tool for graft survival

The “placental model” of tolerance has been directly and successfully applied to the field of solid organ transplantation (55). In this context, HLA-G expression is not a source of pathology but a sign of induced tolerance.

A large body of clinical evidence has demonstrated a strong positive correlation between the expression of HLA-G and improved allograft acceptance (56). This has been observed in heart, kidney, liver, and lung transplant recipients. Higher levels of HLA-G, detected either within the graft tissue via biopsy or, non-invasively, as soluble HLA-G (sHLA-G) in the recipient’s circulation, are associated with a significantly reduced incidence of both acute and chronic rejection episodes (24, 25).

This strong correlation has positioned HLA-G as a promising non-invasive biomarker for monitoring the immune status of transplant patients. Elevated sHLA-G levels may signify a state of operational tolerance and predict better long-term graft survival, potentially allowing for the careful tailoring of immunosuppressive drug regimens (57). Furthermore, these findings have inspired the development of novel therapeutic strategies (discussed in section 5.2.3) aimed at administering HLA-G agonists or inducing its expression to actively promote graft-specific tolerance (58).

### 3.3 Homeostasis in immune-privileged sites: the role of HLA-G in the eye, testis, and other sanctuaries

Beyond the temporary immune-privileged site of the placenta, physiological HLA-G expression is found in a small number of other permanent “sanctuary” tissues that require constitutive protection from immune-mediated damage (59).

The Eye (Cornea): The anterior chamber of the eye and the cornea are classic examples of immune-privileged sites. This privilege is critical for preserving vision, as an inflammatory response in the cornea can lead to scarring and blindness. Studies have confirmed the presence of both HLA-G protein and its transcripts in healthy human corneal tissue, specifically in keratocytes, corneal epithelial cells, and endothelial cells (60). It is widely postulated that this constitutive HLA-G expression, similar to its role in the placenta, is a key factor in maintaining ocular immune tolerance and is a major reason for the high success rate of corneal allografts, which are often performed without HLA matching (59).

The Testis: The testis is another well-established immune-privileged site, essential for protecting developing germ cells from autoimmune attack (61). Sperm-specific antigens appear at puberty, long after central immune tolerance has been established, meaning they are “auto-antigenic.” The blood-testis barrier, formed by Sertoli cells, provides a physical barrier, but an active immunological barrier is also required. HLA-G has been identified in the testis, where its expression by cells like Sertoli and Leydig cells is believed to contribute to the local immunosuppressive microenvironment that actively tolerizes the immune system to the presence of these germ cell antigens (61, 62).

## 4 Pathological roles of HLA-G: immune evasion and disease progression

The potent, multi-faceted tolerogenic program described in the previous section is a double-edged sword(**Figure 1**). The same mechanisms that are essential for physiological tolerance are pathological when aberrantly activated in disease. Malignant tumors and chronic viruses have, through evolutionary pressure, converged on a common strategy: the *de novo* expression of HLA-G. This effectively reactivates the physiological tolerance program in a new, pathological context (16, 63, 64).

This “onco-fetal” or “onco-reproductive” mimicry is a powerful explanatory framework. A tumor expressing HLA-G is, in effect, building its own immune-privileged sanctuary, just as a trophoblast does in the placenta (65). The mechanisms are chillingly parallel: inhibition of local NK and T cells, reprogramming of myeloid cells to a tolerogenic Treg-inducing state and even the facilitation of tissue invasion (64). The HLA-G-driven upregulation of matrix metalloproteinases (MMPs) that enables tumor metastasis is a malignant echo of the same mechanism used by the HLA-G-positive trophoblast to invade the uterine wall (11).

### 4.1 Tumor immune evasion

The most intensively studied pathological role of HLA-G is its aberrant expression in cancer (66). While absent in the vast majority of healthy adult tissues, *de novo* HLA-G expression is detected in a wide variety of human malignancies, including renal cell carcinoma, urothelial bladder carcinoma, melanoma, glioblastoma, colorectal cancer, gastric cancer, and breast cancer (67, 68).

#### 4.1.1 Core mechanisms: inhibiting cytotoxicity, inducing Treg cells, and remodeling the tumor microenvironment

The expression of HLA-G by tumor cells provides a multi-pronged defense against the host immune system.

Direct Inhibition of Cytotoxicity: Tumor-expressed HLA-G acts as a “don’t kill me” signal. By engaging LILRB1 on the surface of infiltrating NK cells and CTLs, it delivers a potent inhibitory signal that directly suppresses their lytic function, allowing the tumor cell to escape destruction (47).

Induction of Regulatory Cells: As detailed in section 2.3, HLA-G expression, particularly through the LILRB2 axis on APCs, is a potent driver of immune suppression. This leads to the generation and expansion of immunosuppressive cell populations within the tumor microenvironment (TME), including myeloid-derived suppressor cells (MDSCs) and, critically, regulatory Treg cells (40). These induced regulatory cells further amplify the immunosuppressive state, inhibiting anti-tumor immune responses.

TME Remodeling and Metastasis: HLA-G expression is not just a defensive shield; it is an offensive weapon for tumor progression. HLA-G has been shown to arm tumor cells with a higher invasive and metastatic potential. This is achieved, in part, by its ability to upregulate the expression of tumor-promoting factors, notably MMPs (69). MMPs are enzymes that degrade the extracellular matrix and basement membrane, a necessary step for local invasion and the entry of tumor cells into the bloodstream to form distant metastases (70). This directly links the immune checkpoint function of HLA-G to the most lethal aspects of cancer progression.

#### 4.1.2 Clinical significance: correlation of HLA-G expression with cancer staging and poor prognosis

The clinical consequence of this multi-pronged immune evasion strategy is a strong and consistent negative correlation between HLA-G expression and patient outcomes (19). In numerous cancer types, HLA-G expression is associated with more advanced tumor stage, higher rates of metastasis, and resistance to therapy.

This observation, long-reported in individual studies, was definitively confirmed by a 2023 systematic review and meta-analysis (19). This study aggregated data from 25 individual studies, encompassing 4,871 patients with various solid tumors. The findings were stark:

HLA-G expression was associated with a significantly worse Overall Survival (OS) across all cancer types.

The combined Hazard Ratio (HR) for mortality was 2.09 (95% Confidence Interval: 1.67–2.63), meaning patients with HLA-G-positive tumors had more than double the risk of death (19).

This negative association was particularly pronounced in gastric cancer (HR = 3.40) and was also highly significant in colorectal cancer (HR = 1.55) (19).

These robust, quantitative data solidify HLA-G’s status as a major negative prognostic biomarker and identify it as a high-priority, clinically-relevant target for cancer immunotherapy (**Table 3**).

**Table 3:** Summary of HLA-G expression and prognostic value in solid tumors.

Cancer type	Key findings (prevalence, staging)	Prognostic impact (OS/DFS)	Hazard ratio (HR) [95% CI]	References
All Solid Tumors (Meta-analysis)	Expression associated with worse survival.	Worse OS	2.09 [1.67–2.63]	(19)
Gastric Cancer	Higher sHLA-G levels *vs*. controls.	Worse survival.	3.40 [1.64–7.03]	(19, 71)
Colorectal Cancer (CRC)	Associated with reduced DFS in some subgroups.	Worse OS.	1.55 [1.16–2.07]	(19)
Pancreatic Cancer	High expression correlated with advanced clinical stage (T stage) and reduced tumor-infiltrating lymphocytes (TILs)	Worse OS.	1.72 [0.79–3.74]	(19)
Breast Cancer	Higher HLA-G expression correlated with fewer tumor-infiltrating lymphocytes (TILs).	Worse outcome.	1.76 [1.15–2.71]	(19)

### 4.2 Autoimmune diseases: HLA-G imbalance and disease activity in SLE, RA, and MS

The role of HLA-G in autoimmune diseases is substantially more complex and less clear-cut than in cancer. Here, the pathology is not one of simple upregulation but rather a dysregulation or imbalance of its tolerogenic function (72). Theoretically, higher levels of the inhibitory HLA-G molecule should be protective against autoimmunity.

However, clinical data are conflicting. Many studies associate the risk of autoimmune diseases like Systemic Lupus Erythematosus (SLE), Rheumatoid Arthritis (RA), and Multiple Sclerosis (MS) not with expression levels per se, but with genetic polymorphisms in the HLA-G regulatory regions (5’ URR and 3’ UTR) (60). This suggests that a genetically-determined “set point” for HLA-G expression may predispose an individual to an imbalanced immune response.

Studies measuring sHLA-G levels in patients have yielded inconsistent results (60). For example, in MS, some research suggests sHLA-G levels in the cerebrospinal fluid (CSF), but not necessarily in the serum, may be a useful marker for monitoring disease activity, while other studies find no association (73). Similarly, for RA, while the HLA-G gene may be upregulated in some patient cells, studies of the key 14-bp polymorphism have been inconclusive (74). This complexity suggests that the role of HLA-G in autoimmunity is highly context-dependent, and a simple “more tolerance is better” model does not fully capture the biology.

### 4.3 Chronic viral infections: exploitation of HLA-G by HIV and HCV for viral persistence

Similar to the strategy employed by tumors, many chronic viruses actively exploit the HLA-G pathway to evade immune clearance and establish a state of persistent infection (75).

The mechanism involves the induction of HLA-G expression on virus-infected host cells. This *de novo* expression, which is not present on the healthy cell, serves as a protective shield. By engaging inhibitory receptors on antiviral NK cells and CTLs, the induced HLA-G suppresses the immune response directed at the infected cell, allowing the virus to replicate and persist (16, 75).

This phenomenon has been documented in:

Human Immunodeficiency Virus (HIV): Elevated levels of sHLA-G are commonly found in the serum of untreated HIV patients. Furthermore, specific HLA-G polymorphisms, such as the G*010108 allele, have been linked to an increased risk of HIV-1 infection, suggesting a genetic predisposition to this evasion mechanism (76).

Hepatitis C Virus (HCV): Patients with chronic HCV infection also show elevated plasma sHLA-G levels (77). In the context of HCV/HIV co-infection, HLA-G expression within the liver is associated with more severe histopathological stages of disease and more rapid progression of liver fibrosis (78). This indicates that HLA-G is not just a marker of infection but an active contributor to its pathology.

### 4.4 Parasitic infections and allergic disorders

Beyond oncology and virology, HLA-G expression plays a nuanced role in parasitic infections and allergic conditions. In parasitic diseases such as Malaria, HLA-G expression is often upregulated as a host-protective mechanism to dampen excessive inflammatory responses that could damage tissues (79). However, similar to the tumor context, parasites may exploit this tolerance to ensure their own survival. For instance, increased soluble HLA-G levels have been observed in patients with Plasmodium falciparum infection, correlating with higher parasitic load but reduced severity of cerebral symptoms (80).

Conversely, in allergic disorders like Asthma and Allergic Rhinitis, where the immune system is hyper-reactive, HLA-G deficiency or downregulation is often observed. Studies suggest that soluble HLA-G levels are significantly lower in asthmatic patients compared to healthy controls, implying that a lack of HLA-G-mediated suppression contributes to the sustained Th2-type inflammation and airway hyper-responsiveness (81). Thus, HLA-G appears to act as a “rheostat” for immune sensitivity, with its absence predisposing individuals to hypersensitivity reactions.

## 5 Translational frontiers: a new target for diagnosis and therapy

Given its central role in both physiological tolerance and pathological evasion, the HLA-G axis has become one of the most exciting and rapidly developing frontiers in translational medicine (82).

### 5.1 HLA-G as a diagnostic and prognostic biomarker

The strong correlation between HLA-G expression and disease status has driven significant research into its use as a clinical biomarker.

#### 5.1.1 Soluble HLA-G as a liquid biopsy metric

The soluble isoforms of HLA-G (primarily HLA-G5 secreted from the cell and HLA-G1 shed from the membrane) can be detected and quantified in peripheral blood (serum or plasma) (83). This makes sHLA-G an ideal candidate for a non-invasive “liquid biopsy.” Elevated sHLA-G levels have been reported in patients with various cancers, including gastric cancer and melanoma, when compared to healthy control groups (84). However, its utility as a prognostic marker, when measured as a total pool, has been debated, with some studies showing inconsistent results and limitations (85).

#### 5.1.2 Emerging hotspot: exosome-mediated HLA-G transfer and its diagnostic potential

A critical and recent refinement in the field is the differentiation between free sHLA-G (likely monomeric/dimeric HLA-G5) and HLA-G that is transported via extracellular vesicles (EVs), particularly exosomes. Tumor cells are known to package and release membrane-bound proteins (like HLA-G1) on the surface of exosomes (86).

This distinction is not merely technical; it appears to be of profound biological and prognostic importance. Strikingly, several studies, particularly in breast and ovarian cancer, have found that it is exclusively the HLA-Gev (exosomal) fraction, and not the free sHLA-G fraction, that correlates with poor prognosis and disease progression (87). In one study, high levels of free sHLA-G were paradoxically associated with longer survival, while high HLA-Gev was associated with poor outcomes, highlighting their distinct biological roles (88).

This finding reveals a more insidious and potent mechanism of immune evasion. A free sHLA-G molecule may have a diffuse, localized, or transient effect. An exosome, however, is a stable, targeted delivery vehicle for long-range intercellular communication. A tumor cell releasing HLA-Gev is not just defending itself locally; it is “pre-suppressing” the entire immune system at a distance (89). These exosomes can travel to distal sites, such as lymph nodes, and “tolerize” APCs before they have a chance to prime anti-tumor T cells, or neutralize NK cells in the circulation before they can extravasate to the tumor (90). This represents a proactive and systemic immune evasion strategy, which explains its strong correlation with advanced disease and poor prognosis.

### 5.2 Therapeutic STRATEGIES TARGETING THE HLA-G/ILT axis

The identification of the HLA-G/ILT axis as a major, non-redundant immune checkpoint has triggered a wave of drug development, positioning it as a highly promising, next-generation therapeutic target (71).

#### 5.2.1 Cancer immunotherapy: blockade of the HLA-G/ILT axis (antagonists)

For cancer, the therapeutic goal is to block the inhibitory HLA-G/ILT signal, thereby “releasing the brakes” on the anti-tumor immune response (91). Several distinct strategies are now in clinical development to achieve this:

Targeting the Ligand (HLA-G): This approach uses a monoclonal antibody to bind directly to the HLA-G molecule on the tumor cell, preventing it from engaging any of its receptors.

Drug Candidate: TTX-080 (Tizona/Gilead). This is a first-in-class antibody designed to bind to HLA-G and block its interaction with both LILRB1 and LILRB2, thus simultaneously “waking up” T/NK cells and myeloid cells (14).

Targeting the Receptors (LILRB1 and LILRB2): Given that LILRB1 and LILRB2 are the primary effectors of HLA-G-mediated suppression, blocking these receptors offers a direct strategy to restore immune competence. LILRB1 Blockade: BND-22 (SAR444881) is a prominent anti-LILRB1 antibody currently in Phase 1/2 trials. It selectively blocks the interaction of LILRB1 with HLA-G and classical MHC-I, thereby enhancing the cytotoxic activity of NK and T cells (92). In addition to BND-22, other novel LILRB1 antagonists are under clinical evaluation. AGEN1571 is a high-affinity antibody that has shown potential to promote adaptive and innate immune responses in preclinical models (93). Similarly, ADA-011, a humanized anti-LILRB1 monoclonal antibody, is being tested in Phase 1 trials as a monotherapy or in combination with anti-PD-L1 agents for advanced solid tumors (93).

LILRB2 Blockade: LILRB2 (ILT4) is critically involved in myeloid cell suppression and the reprogramming of the tumor microenvironment (TME). Several antibodies targeting this receptor have entered clinical development. MK-4830 represents a leading candidate; in Phase 1 trials, it demonstrated the ability to reprogram suppressive myeloid cells and showed durable objective responses in heavily pre-treated patients (93). JTX-8064 is another LILRB2 inhibitor designed to switch macrophages from a suppressive to a pro-inflammatory phenotype, currently in Phase 1/2 trials (93). Other candidates include IO-108, which has shown promise in activating cytotoxic T lymphocytes and APCs, and ES009, which is being evaluated for its safety and ability to potentiate T cell activation (93).

Dual and Multi-Target Strategies: To overcome redundancy, next-generation therapies are targeting multiple checkpoints simultaneously. NGM707 is a dual antagonist antibody targeting both LILRB1 and LILRB2, aiming to broadly reverse myeloid and lymphoid suppression. IOS-1002 takes this further by targeting LILRB1, LILRB2, and KIR3DL1, showing potent efficacy in preclinical models (93). Furthermore, bispecific antibodies are emerging, such as CDX-585, which targets both PD-1 and LILRB2 to simultaneously inhibit the PD-1 axis and reprogram myeloid cells.

Forcing the Interaction (Bispecific T-cell Engager): This is the most aggressive approach, turning the inhibitory signal into a “kill” signal. Drug Candidate: JNJ-78306358 (Janssen). This is a bispecific T-cell engager (BiTE) antibody. One arm of the antibody binds to HLA-G on the tumor cell, while the other arm binds to CD3 on the T cell. This physically tethers the T cell to the tumor cell, forcing T-cell activation and targeted killing, irrespective of the T cell’s original specificity (**Table 4**) (95).

**Table 4:** Clinical development of therapeutics targeting the HLA-G/ILT axis.

Drug candidate	Target	Mechanism of action (MoA)	Therapy type	Key clinical trial(s)	References
TTX-080	HLA-G (Ligand)	Anti-HLA-G mAb. Blocks HLA-G from binding to LILRB1 and LILRB2.	Checkpoint Inhibitor (Antagonist)	NCT04485013	(14, 16)
BND-22 (SAR444881)	LILRB1 (Receptor)	Anti-LILRB1 mAb. Blocks LILRB1 interaction with HLA-G and classical MHC-I.	Checkpoint Inhibitor (Antagonist)	NCT04717375 NCT06651593	(92, 94)
JNJ-78306358	HLA-G x CD3 (Bispecific)	Bispecific T-cell Engager. Binds HLA-G on tumor and CD3 on T cell.	T-Cell Redirector (Activator)	NCT04991740	(16, 95)
AGEN1571	LILRB1 (Receptor)	Novel high-affinity anti-LILRB1 mAb. Promotes adaptive and innate immune responses.	Checkpoint Inhibitor (Antagonist)	NCT05377528 (Phase 1)	(93)
ADA-011	LILRB1 (Receptor)	Humanized anti-LILRB1 mAb. Tested alone or with anti-PD-L1.	Checkpoint Inhibitor (Antagonist)	NCT05061219 (Phase 1)	(93)
MK-4830	LILRB2 (Receptor)	Anti-LILRB2 (ILT4) mAb. Reverses myeloid suppression and enhances T cell response.	Checkpoint Inhibitor (Antagonist)	NCT03564691 (Phase 1)NCT05446870 (Phase 2)	(93)
JTX-8064	LILRB2 (Receptor)	Anti-LILRB2 mAb. Reprograms macrophages to drive T-cell activation.	Checkpoint Inhibitor (Antagonist)	NCT04669899 (Phase 1/2)	(93)
IO-108	LILRB2 (Receptor)	Fully human anti-LILRB2 mAb. Promotes innate and adaptive anti-cancer immunity.	Checkpoint Inhibitor (Antagonist)	NCT05054348 (Phase 1)	(93)
NGM707	LILRB1 & LILRB2	Dual antagonist mAb targeting both LILRB1 and LILRB2.	Dual Checkpoint Inhibitor	NCT04913337 (Phase 1/2)	(93)
IOS-1002	LILRB1, LILRB2, KIR3DL1	Triple target inhibitor. Increases anti-tumor responses of macrophages, T and NK cells.	Multi-target Inhibitor	NCT05763004 (Phase 1)	(93)
LILRB-based CAR-T	HLA-G+ Tumor Cells	Engineered T-cells expressing LILRB1/2 extracellular domains identify and eliminate HLA-G-expressing tumor cells.	Cell Therapy (CAR-T)	Preclinical	(96)
Exosome Depletion / Inhibition	HLA-Gev (Exosomes)	Blocking exosome secretion, physical removal via apheresis, or utilizing exosomes as drug delivery vectors.	Exosome-Targeted Therapy / Apheresis	Preclinical / Exploratory	(88, 90)

#### 5.2.2 Emerging hotspot: combination therapy with PD-1/PD-L1 inhibitors

A critical strategy moving forward is the combination of HLA-G blockade with existing checkpoint inhibitors, such as anti-PD-1 (e.g., pembrolizumab, nivolumab) or anti-PD-L1 (e.g., atezolizumab) (67).

The scientific rationale for this is compelling. HLA-G and PD-L1 represent two parallel and non-redundant inhibitory pathways that are often co-expressed by tumors (94). A major clinical problem is that many patients are or become resistant to anti-PD-1 therapy (97). This resistance may be driven by the compensatory upregulation of other checkpoints, with HLA-G being a prime candidate. A tumor being suppressed by anti-PD-1 treatment may simply “shift” its immune defense to the HLA-G axis.

Therefore, by blocking both pathways simultaneously, it is hypothesized that a much deeper, more complete, and more durable reversal of immune exhaustion can be achieved (14). This synergistic strategy is the explicit goal of the NCT04485013 clinical trial, which is testing the anti-HLA-G antibody TTX-080 in combination with the anti-PD-1 antibody pembrolizumab in patients with advanced solid tumors.

#### 5.2.3 Tolerance induction: HLA-G agonists and engineered cells for transplantation and autoimmunity

For non-cancer pathologies, the therapeutic goal is inverted. Instead of blocking HLA-G, the aim is to enhance its tolerogenic signaling to treat conditions of unwanted immune activation (98).

In Transplantation: The observation that sHLA-G correlates with graft survival has led to pre-clinical development of recombinant HLA-G agonists. Administering recombinant soluble HLA-G5 has shown promise in animal models for promoting graft acceptance and inducing allograft-specific tolerance, potentially reducing the need for toxic, broad-spectrum immunosuppressants (58, 99).

In Autoimmunity: For autoimmune diseases, future therapies may involve the administration of HLA-G agonists or the use of engineered cell therapies. One advanced concept is the ex vivo generation of tolerogenic dendritic cells (DC-10). These are a specific subset of DCs known to secrete high levels of IL-10 and, critically, express high levels of HLA-G. These engineered DC-10s are potent inducers of regulatory Tr1 cells and could, in theory, be infused into a patient to restore immune homeostasis (100).

#### 5.2.4 Emerging Modalities: CAR-T and exosome-targeted therapies

Recent advances have expanded the therapeutic landscape beyond monoclonal antibodies. Chimeric Antigen Receptor T-cell (CAR-T) therapy is being adapted to target the HLA-G axis. Preclinical studies are evaluating CAR-T cells engineered with an extracellular domain derived from LILRB1 or LILRB2, allowing them to specifically recognize and eliminate HLA-G-expressing tumor cells and suppressive stromal cells (96). This approach aims to overcome the “immune desert” often created by HLA-G in solid tumors.

Furthermore, the discovery that HLA-G-bearing exosomes (HLA-Gev) are potent mediators of systemic immune suppression has spurred interest in exosome-targeted therapies. Strategies are being developed to either block the secretion of these exosomes or to remove them from circulation via apheresis-like techniques (87, 89). Additionally, because HLA-G+ exosomes specifically home to tumor sites, researchers are exploring their potential as natural delivery vectors for chemotherapeutic drugs or RNA interference (RNAi) agents, turning a tumor’s evasion mechanism into a Trojan horse for its destruction (96).

## 6 Conclusion and future perspectives

The journey of HLA-G from an obscure placental protein to a next-generation immune checkpoint target has been remarkable. It is now clear that HLA-G is a master regulator of the immune system, whose dual-role as a mediator of physiological tolerance and a driver of pathological evasion makes it a target of immense clinical importance. However, as the field races toward clinical translation, it is encumbered by significant and persistent challenges that must be addressed (11).

### 6.1 Current challenges: detection standardization, functional polymorphism, and *in vivo* complexity

Despite decades of progress, the single greatest hurdle impeding the clinical maturation of HLA-G is the crisis in detection and standardization (101).

#### 6.1.1 Antibody specificity

A vast swath of the published literature on HLA-G expression is built on data from the 4H84 monoclonal antibody. It is now known that this antibody exhibits significant cross-reactivity with classical HLA-I molecules, especially HLA-A, -B, and -C (102). This means that many studies may have systematically overestimated the prevalence of HLA-G, and some “HLA-G positive” findings may have been false positives.

#### 6.1.2 Isoform blindness

The field is operating with a critical lack of tools. The vast majority of available antibodies are “pan-HLA-G” and cannot distinguish between the seven functionally distinct isoforms (11). A simple “HLA-G positive” result from an immunohistochemistry stain is an ambiguous and largely uninformative data point. It is clinically vital to know which isoform is present: Is it the full-length membrane-bound HLA-G1? The secreted HLA-G5? A truncated, perhaps non-functional, variant? Or is it the prognostically-critical HLA-Gev? Without isoform-specific quantification, data interpretation is severely hampered.

#### 6.1.3 Lack of universal procedures

The literature is rife with methodological inconsistencies. Studies use different antibodies (many with unverified specificity), different scoring methods, and different arbitrary cut-off values for “positivity” (e.g., 5% *vs* 50% of cells) (102). This makes meaningful cross-study comparison and meta-analysis exceptionally difficult and unreliable.

Beyond these technical challenges lie deep biological questions. The precise *in vivo* functional consequences of the different regulatory polymorphisms (like the 14-bp ins/del) are still not fully resolved (103). The functional differences between monomers, dimers, and multimers, and their roles in different biological compartments (cell surface *vs*. exosome *vs*. free soluble), remain a complex and poorly understood field (16).

### 6.2 Future directions: HLA-G as a next-generation checkpoint for precision medicine

To unlock the full potential of HLA-G, the field must pivot from its current exploratory phase to one defined by rigorous standardization and mechanistic precision.

The immediate and urgent priority is the development and validation of a new generation of isoform-specific monoclonal antibodies and standardized, quantitative assays (11). These tools must be capable of accurately and reproducibly quantifying each of the key isoforms (e.g., G1, G5) and, critically, distinguishing between free sHLA-G and HLA-Gev in liquid biopsies.

With these tools, future research can definitively dissect the *in vivo* function of each isoform and the true clinical impact of the various regulatory polymorphisms. Alternative therapeutic strategies, such as small molecules that prevent HLA-G dimerization or target its peptide-binding groove, should also be explored (104).

The ultimate goal is to move HLA-G from a general prognostic marker to a predictive biomarker that guides precision medicine. A future clinical workflow might involve:

Genotyping the patient’s HLA-G 5’ URR and 3’ UTR to determine their baseline expression potential.

Quantifying isoform-specific HLA-G, particularly HLA-Gev, in their plasma as a real-time measure of tumor immune evasion.

Profiling the TME for co-expression of LILRB1, LILRB2, and PD-L1.

Using this multi-modal signature, clinicians could one day stratify patients to select the optimal, individualized therapy: anti-HLA-G (TTX-080) for one patient, anti-LILRB1 (BND-22) for another, a bispecific T-cell engager for a third, or a combination blockade with anti-PD-1 for a fourth. Only by resolving the fundamental complexities of HLA-G biology can we fully realize its potential as a transformative target in immunology.
